# Clinical and Aberrometric Evaluation of a New Monofocal IOL with Intermediate Vision Improvement

**DOI:** 10.1155/2022/4119698

**Published:** 2022-07-07

**Authors:** Alessandro Bova, Stefano Vita

**Affiliations:** Eye Clinic, Department of Medicine, Surgery and Health Sciences, University Hospital of Monfalcone, Monfalcone 34074, Italy

## Abstract

**Purpose:**

The aim of the study was to evaluate the visual outcomes, aberrometric results, and subjective and objective optical qualities 12 months after implantation of a new monofocal intraocular lens (Physiol IsoPure 1.2.3) in comparison with a standard monofocal intraocular lens (Tecnis PCB00).

**Materials and Methods:**

Cataract patients without ocular comorbidities had bilateral implantation of the IsoPure IOL or the PCB00 IOL. One month after eye surgery, the visual acuity and monocular defocus curve were assessed. Twelve months after surgery, the visual acuity, binocular defocus curve, contrast sensitivity, and subjective/objective optical quality were assessed. Furthermore, wavefront analysis was performed. The primary endpoint was the best distance correct/uncorrected visual acuity at intermediate and far distances. The secondary endpoint was an aberration evaluation of the IOLs and contrast sensitivity.

**Results:**

The study comprised a total of 42 patients (84 eyes). Monocular and binocular uncorrected and corrected distance were similar between groups, and uncorrected intermediate visual acuity was significantly higher in the IsoPure group. There was no difference in contrast sensitivity and subjective and objective optical qualities. The optical aberrations at 3.0 and 5.0 mm aperture diameters were similar in both groups.

**Conclusion:**

The IsoPure IOL, based on greater depth of focus than the aspheric monofocal IOL, may offer a good option for the distance and intermediate vision without increasing optical aberrations and any photic phenomena.

## 1. Introduction

Today, the intermediate vision has become more important than ever because of the increase use of phones, computers, and tablets in people's daily life but also for shopping, applying makeup, and playing cards. Thus, the expectations of patients following cataract surgery are rising [1]. In the standard treatment of cataract, conventional monofocal intraocular lenses (IOLs) do not address intermediate vision [2]. Monofocal IOLs provide a single point of focus for far vision, making reading glasses essential. The multifocal IOLs reduced spectacle dependence after surgery but sometimes patients complain of optical side effects, such as decreased contrast sensitivity, glare or halos, and inadequate intermediate vision [3, 4]. To overcome these issues, it is growing interest toward IOLs that may reduce these unwanted effects.

The monofocal, Physiol IsoPure 1.2.3, IOL has been developed to allow a good distance vision as well as aspherical lenses and improves, unlike them, the intermediate vision, developing a new segment in the market of IOLs.

The IsoPure IOL is based on a technology that aims to improve intermediate visual acuity by extending the depth of focus (EDOF), without inducing photic effects. The optic is monofocal combined with a unique design of the IOL surface.

This study aimed to evaluate the late findings of the implanted (Physiol IsoPure 1.2.3) IOL in patients who underwent cataract surgery and to compare the outcomes with a standard monofocal aspheric IOL (Tecnis PCB00).

## 2. Materials and Methods

### 2.1. Study Participants

The present study assessed the visual acuity and aberrometric results in two groups of patients who had bilateral implantation of the IsoPure IOL or the monofocal PCB00 IOL.

Eighty-four eyes of 42 patients underwent cataract surgery from 2020 to 2021 at the Monfalcone Eye Clinic. Patients with moderate cataract and corneal astigmatism less than 1 diopter were included in the study. The eyes were divided into two groups: Physiol IsoPure IOL (42 eyes) and Tecnis PCB00 IOL (42 eyes).

This is a single-center, retrospective study, and all patients were informed about the study and provided fully informed consent to permit the usage of their data in the study. The study was approved by the local regulative committee and followed the tenets of the Declaration of Helsinki.

### 2.2. Intraocular Lenses

The IsoPure 1.2.3 IOL (PhysIOL, Liege, Belgium) is an acrylic hydrophobic glistening-free lens (G-free) with four closed haptics, and it has an ultraviolet and light blue filter, a total diameter of 11.0 mm, and an optic diameter of 6.0 mm. The optical A-constant is 119.4. The IsoPure 1.2.3 IOL is a monofocal lens that combines an anterior and posterior surface profile of increased negative spherical aberration and fine-tuned for each diopter on the whole [5].

The Tecnis PCB00 IOL (Johnson and Johnson Vision, AMO Groningen BV) is a monofocal acrylic hydrophobic aspheric lens with an ultraviolet filter, a total diameter of 13.0 mm, and an optic diameter of 6.0 mm. The optical A-constant is 119.3 [6].

### 2.3. Surgical Technique

All patients were operated on by the same surgeon (SV). The main incision was made superiorly for 2.2 mm. Following a standard phacoemulsification surgery, the preloaded monofocal IOLs were implanted into the capsular bag. Topical antibiotics and corticosteroids were administered to all patients four times a day for two weeks, and topical nonsteroidal anti-inflammatory drugs were administered three times a day for 40 days.

### 2.4. Postoperative Evaluation

Measurements were taken one month and twelve months after eye surgery. Monocular uncorrected (UDVA) and corrected (CDVA) distance visual acuities, monocular uncorrected intermediate visual acuities (UIVAs), and distance-corrected intermediate visual acuities (DCIVAs) were measured at 1 month, binocular and monocular UDVAs/CDVAs were measured at 12 months using an ETDRS study chart, and UIVAs/DCIVAs were measured at 12 months using optotypes (precision vision). UIVA and DCIVA were measured at 66 cm using optotypes because these distances are the preferred distance for viewing laptops or tablet devices. Defocus curves were obtained with monocular vision at 1 month and with binocular vision at 12 months by adding plus lenses (up to +1.0 D) and minus lenses (up to −2.0 D) in 0.5 D steps to the distance optical correction and then recording the visual acuity with each lens power.

Contrast sensitivity was measured with binocular vision and without correction. The computer-displayed vision chart CSO (Costruzione Strumenti Oftalmici, Florence, Italy) presents sine-wave gratings at 1.5, 3.0, 6.0, 12.0, and 18.0 cycles per degree (cpd) with a background luminance of 85 candelas/m^2^. The mean of the log10 values for each spatial frequency was used for comparison.

Optical aberrations were assessed with Osiris (CSO, Florence Italy), a high-resolution pyramidal wavefront sensor-based aberrometer. Evaluated aberrations were ocular, corneal, internal HOAs, and the following Zernike (Z) coefficients: Z3(−1, +1), which represents coma aberration; Z3(−3, +3), which represents trefoil aberration; and Z(4, 0), which represents spherical aberration. Measurements were taken in mydriasis for 3.0 mm and 5.0 mm aperture diameters.

The point-spread function derived from the optical HOAs and expressed as the Strehl ratio was taken as an objective indicator of the postoperative optical quality of the eyes [7].

The subjective optical quality was assessed using the Visual Function Index-14 (VF-14) questionnaire, which asked patients how much difficulty they had with routine activities such as cooking and playing cards and also using a questionnaire that asked patients whether they had visual disturbances at night such as halos, glare, starbursts, and hazy vision.

### 2.5. Statistical Analysis

Data analysis was performed using the MS Excel 2020 software (Microsoft Corporation, Redmond, Washington, USA) and SPSS for Windows version 15.0 (IBM, Armonk, NY, USA).

We calculated that to detect a clinically significant difference of 0.1 logarithm of the minimum angle of resolution (logMAR) in the binocular uncorrected intermediate visual acuities at 12 months postoperatively with 80% power (*α*=0.05), 17 patients per group would have been necessary.

Data samples were evaluated using the Kolmogorov–Smirnov test and student's *t*-test for the comparison between groups. A *p* value of less than 0.05 was considered statistically significant for all tests.

## 3. Results and Discussion

### 3.1. Results

We enrolled 42 eyes in the IsoPure group and 42 eyes in the PCB00 group. In the IsoPure group, there were 9 males and 12 females, while in the PCB00 group there were 11 males and 10 females. The mean age of patients in the IsoPure group was 71.33 ± 7.91 years and 70.11 ± 6.51 years in the PCB00 group. The mean axial length in the IsoPure group was 23.74 ± 0.33 and 23.50 ± 0.45 mm in the PCB00 group (*p*=0.81). The preoperative spherical equivalent was +0.78 ± 1.91 in the IsoPure group and +0.73 ± 2.05 in the PCB00 group (*p*=0.62), and corneal astigmatism was less than 0.75 D in each group (*p*=0.69). The other clinical characteristics of the patients are shown in Table 1. There was no significant difference in the preoperative parameters between both groups.

No patient had a posterior capsular opacification that needed Nd, YAG laser capsulotomy, or any others postoperative cataract complications.

Tables 2 and 3 summarize the postoperative visual acuity one month and twelve months after cataract surgery: IsoPure and PCB00 had similar acuity at distance (4 m) twelve months after cataract, and there was no significant difference in the postoperative binocular UDVA (*p*=0.89) and CDVA (*p*=0.90) in both groups. The IsoPure group had a significantly better monocular and binocular uncorrected intermediate visual acuity (UIVA) than to the PCB00 group (*p* < 0.01).

**Figure 1:**
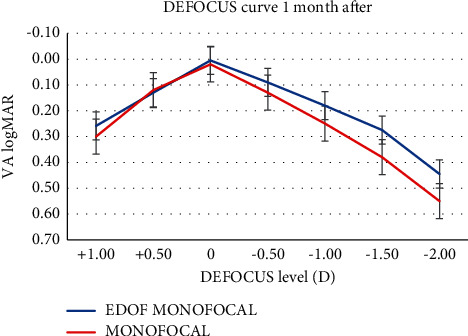
The defocus curve obtained monocularly with uncorrection (EDOF: extended depth of focus; logMAR: logarithm of the minimum angle of resolution; VA: visual acuity).

**Figure 2:**
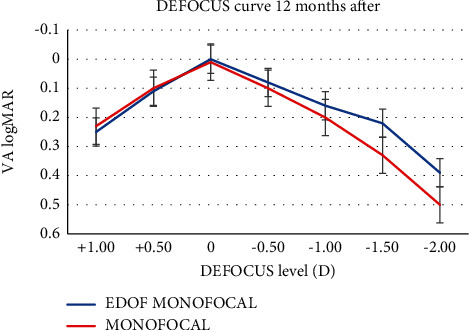
The defocus curve obtained binocularly with uncorrection (EDOF: extended depth of focus; logMAR: logarithm of the minimum angle of resolution; VA: visual acuity).

Figure 1 shows the defocus curves obtained with monocular vision 1 month after surgery, and Figure 2 shows binocular vision 12 months after surgery. The defocus curve measured in two groups shows a similar profile from +0.50 D to −0.50 D, with a peak at 0 D defocus, and a reduction in visual acuity with the increase in negative defocus. However, the IsoPure IOL's negative defocus curve was smoother than that of the PCB00 IOL, with a wider landing zone especially within the intermediate defocus levels. A binocular CDVA of 0.10 logMAR or better was maintained between +0.50 D and −0.75 D of defocus in the EDOF group and between +0.50 D and −0.50 D in the monofocal group. For −2.00 D defocus, visual acuity was 0.38 ± 0.05 logMAR in the IsoPure group and 0.50 ± 0.06 logMAR in the monofocal group; the mean difference of 0.12 logMAR was considered as clinically significant (*p* < 0.05). The difference in visual acuity between the IsoPure group and the monofocal group was statistically significant from −1.00 D to −2.00 D of defocus (Table 4).

**Figure 3:**
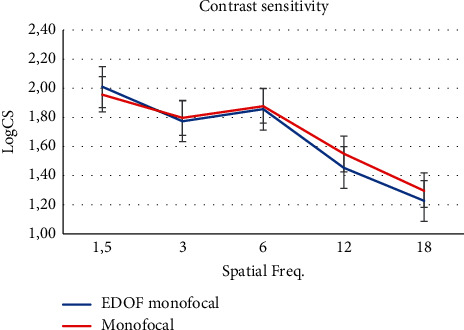
Binocular contrast sensitivity.

Figure 3 shows the logarithm of the contrast sensitivity values at different spatial frequencies. There was no significant difference between groups for any spatial frequency as shown in Table 5 (*p* > 0.05 for all comparisons).

The VF-14 questionnaire (Table 6) demonstrated that patients with bilateral IsoPure implantation have greater independence in the activity that requires intermediate vision (playing cards, cooking, reading large print, and distinguishing people close up). None of the patients implanted with the IsoPure IOL described a night dysphotopsy, halo, or glare, and they were very satisfied.

The mean optical quality, evaluated using the Strehl ratio, at a 3.0 mm aperture diameter was 0.25 ± 0.13 in the EDOF monofocal group and 0.23 ± 0.11 in the monofocal group (*p*=0.49), at a 5.0 mm aperture diameter was 0.14 ± 0.09 in the EDOF monofocal group and 0.16 ± 0.06 in the monofocal group (*p*=0.51). There was no significant difference between groups.

**Figure 4:**
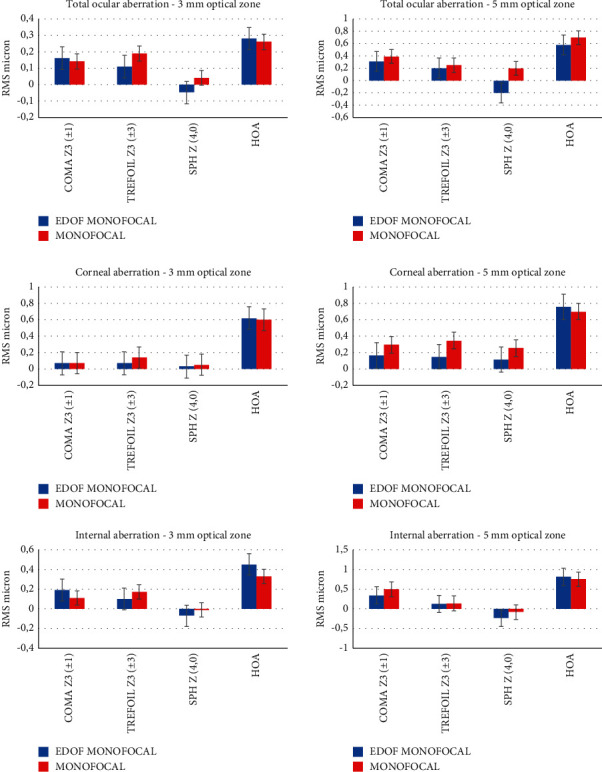
Higher-order aberrations at the 3.0 mm and 5.0 mm aperture diameter (EDOF: extended depth of focus; HOAs: total higher-order aberrations; RMS: root mean square; SPH: spherical aberration).

Figure 4 shows the total ocular, corneal, and internal aberrations measured at 3.0 mm and 5.0 mm aperture diameters. The coma, spherical aberration, and higher-order aberration change on different pupil sizes but were similar between the groups (*p*=0.45). The ocular coma was similar between two groups (*p*=0.58) There were some differences in the internal spherical aberration Z(4, 0) at a 3.0 and 5.0 aperture diameter, more negative in the IsoPure IOL but not statistically significant (*p*=0.024). Although aberrations in the entire eye were not significantly different between the groups at the 3.0 and 5.0 aperture diameter (*p*=0.45 for all comparisons).

### 3.2. Discussion

Daily life has changed compared to the past, and the intermediate vision has become very important to use tablets, computers, and having hobbies [8].

Cataract surgery is a highly successful procedure, and the most used lenses are monofocal IOLs that provide excellent results in distance vision but cannot improve intermediate visions [9,10].

At the same time, implantation of bifocal, trifocal, or extended range of vision IOLs can offer very good visual outcomes without spectacles at different distances [4, 11], but the choice of these IOLs premium depends on patients' characteristics, visual expectations, and preferences. Nevertheless, these IOLs can cause halo or glare perception, a decrease in retinal image quality, which might not be well tolerated by all patients [12]. And, at times, logistical and/or economic limitations can preclude the implantation of a premium IOL. To overcome these unwanted problems, it is developing a new segment in the market of monofocal IOLs.

The IsoPure IOL is based on a technology that aims to improve intermediate visual acuity by extending the depth of focus, without inducing photic effects or loss of visual quality. The IOL design is characterized by smooth and progressive changes in the superficial geometry and incorporates a modification of spherical aberration, known as isofocal technology. The isofocal technology combines a monofocal refractive optic with a polynomial complex surface design of high-order conical surfaces with the right balance of spherical aberrations, that is, fine-tuned for each diopter on the whole optic. This multiconfiguration optimized the optical quality in a range of focus. The design allows central and peripheral light rays not to converge identically, like a monofocal IOL, but instead the plane of the best image shifts between the different foci and over the retina causing an extension of the light wavefront on the retina. When the pupil constricts, the focus of the peripheral rays is no longer visible, the plane of the best image is then formed with the only rays reaching the retina and the eye becomes slightly myopic. An elongated depth of the field of around 1 diopter is achieved, and thus, intermediate vision improves; this represents an increase of around 50% compared to a standard aspheric monofocal IOL [13].

In the present study, the excellent postoperative uncorrected distance was obtained with both IOLs, but the Isopure IOL provided a significantly better UIVA and DCIVA than the standard monofocal IOL (*p* < 0.01). A similar result was obtained by Mencucci et al. [14] with the ICB00 Eyhance IOL, a monofocal IOL that slightly extends the intermediate range by a central 1 mm zone of increased curvature [15].

Regarding the defocus curve, the IsoPure IOL's defocus curve was flatter than the PCB00 IOL's defocus curve, especially within the intermediate defocus levels, with a binocular CDVA of 0.20 logMAR or better maintained between +0.50 D and −1.50 D of defocus. A similar defocus curve was obtained with the ICB00 Eyhance IOL in the previous study [14,15] that showed that the ICB00 provided a visual acuity equal or better than 0.22 logMAR between defocus levels of +1.00 and −1.50 D.

We studied optical aberrations to determine the effect of the IsoPure IOL on the postoperative aberration profile in patients' eyes. The values of total coma and total sphere are comparable with data reported in other studies [16–18]. At 5.0 mm, we found a little increase in negative spherical aberrations with the IsoPure IOL compared with the aspheric monofocal IOL, but the IsoPure IOL did not increase the optical aberrations of the whole eye at 3.0 mm or 5.0 mm, and SA was not statistically significant between two groups (*p*=0.22). The surface design with the negative pericentral spherical aberration is the probable reason for this finding. Optical bench testing showed that the lsoPure IOL reaches a total negative spherical aberration (−0.07 *μ*m) with a pupil aperture of 3.0 mm and a frequency of 50 cycles/mm. This level of negative spherical aberration is sufficient to generate a depth of focus of 0.8 D in a 3 mm pupil, representing an increase in the depth of focus compared to the standard aspheric monofocal IOL [5]. This was the first clinical study exploring optical aberrations of the monofocal IOL that slightly extends the intermediate range.

The postoperative optical quality measured using the Strehl ratio in our study was similar between the two groups and reflects the no statistically significant difference in the HOAs.

The binocular contrast sensitivities, evaluated using the sinusoidal grating method, were similar in both groups at each different spatial frequencies and inside the normal area. Furthermore, none of the patients implanted with the IsoPure IOL described a night dysphotopsy, halo, or glare. In a previous study [19], a similar contrast sensitivity level under photopic and mesopic conditions was obtained with the ICB00 Eyhance IOL. Like a monofocal IOL, IsoPure uses all the available light energy to extend the range of focus. It does not lose light energy through diffraction like multifocal IOLs, and through this design, it maintains contrast sensitivity comparable to a monofocal IOL. Thus, this lens may be offered to the patients who wear multifocal lenses (multifocal or trifocal) might not be well tolerated, such as drivers or a meticulous person that do not accept possible alterations in visual quality but will not provide comparable near vision or spectacle independence for near distances.

Stodulka and Slovak [20] recently published the clinical outcomes of patients implanted with the IsoPure IOL. Their clinical study confirmed the predicted gain at intermediate and comparable visual acuity at distance and normal contrast sensitivity. These clinical outcomes confirm the results reported in our study and, therefore, further validate the methodology herein used.

Carones et al. revealed that the extended depth of focus IOL had better tolerance to residual refractive errors in comparison with bifocal and trifocal IOLs [21]. In another study, Son et al. showed that the EDOF IOL group had better-uncorrected distance visual acuity than the monofocal lens group for the unexpected postoperative residual refractive errors like spherical equivalent values of +0.50 D or −0.50 D [22]. Stodulka and Slovak [20] published the visual acuity obtained with the IsoPure IOL after the induction of different values of the positive and negative cylinder and showed that it dependent on the axis, with superior vision at an axis 180° and similar results at axis of 90° in comparison to the multifocal IOLs. Residual cylinders from +1.0 D to −1.25 D should therefore have no significant impact on visual acuity (better than 0.2 logMAR), and patient satisfaction and the lens may present an advantage.

In this preliminary study, the follow-up of 12 months is a long period that allows us to confirm the great IOL performance at distance and intermediate visual acuity.

## 4. Conclusion

The IsoPure isofocal IOL, based on a greater depth of focus than the aspheric monofocal IOL, may offer a good option for the distance and intermediate vision with minimal reduction of contrast sensitivity, without increasing optical aberrations regardless of a pupil diameter and without causing any photic symptoms.

## Figures and Tables

**Table 1:** Preoperative patient characteristics.

Parameter	IsoPure 1.2.3	PCB00	*p* value
Age (y)	71.33 ± 7.91	70.11 ± 6.51	0.76
Preop CDVA (logMAR)	0.43 ± 0.13	0.41 ± 0.14	0.51
Axial length (mm)	23.74 ± 0.33	23.50 ± 0.45	0.81
Flat K (D)	43.57 ± 0.80	43.71 ± 0.70	0.69
Steep K (D)	44.09 ± 0.54	44.16 ± 0.47	0.76
Pupil diameter (mm)	3.04 ± 0.49	3.02 ± 0.35	0.77
ACD (mm)	3.03 ± 0.33	3.03 ± 0.40	0.84
SE (D)	+0.78 ± 1.91	+0.73 ± 2.05	0.62
Astigmatism (D)	−0.48 ± 0.25	−0.52 ± 0.28	0.69

CDVA (corrected distance visual acuity); logMAR (logarithm of the minimum angle of resolution); K (keratometry); ACD (anterior chamber depth); SE (spherical equivalent).

**Table 2:** One month postoperative monocular visual outcomes.

Parameter	IsoPure 1.2.3 *N* = 42	PCB00 *N* = 42	*p* value
UDVA	0.02 ± 0.03	0.03 ± 0.02	0.81
CDVA	0.01 ± 0.03	0.01 ± 0.05	0.88
UIVA (66 cm)	0.22 ± 0.10	0.39 ± 0.09	<0.001
DCIVA (66 cm)	0.20 ± 0.09	0.37 ± 0.08	<0.001

UDVA = uncorrected distance visual acuity; CDVA = corrected distance visual acuity; UIVA = uncorrected intermediate visual acuity; DCIVA = distance-corrected intermediate visual acuity.

**Table 3:** Twelve month postoperative visual outcomes.

Parameter	IsoPure 1.2.3 *N* = 42	PCB00 *N* = 42	*p* value
UDVA monocular	0.04 ± 0.05	0.05 ± 0.06	0.74
UDVA binocular	0.03 ± 0.04	0.03 ± 0.06	0.89
CDVA monocular	0.03 ± 0.05	0.03 ± 0.04	0.88
CDVA binocular	0.01 ± 0.03	0.01 ± 0.02	0.90
UIVA monocular (66 cm)	0.24 ± 0.11	0.38 ± 0.11	<0.001
UIVA binocular (66 cm)	0.22 ± 0.06	0.33 ± 0.07	<0.001
DCIVA monocular (66 cm)	0.23 ± 0.07	0.36 ± 0.07	<0.001
DCIVA binocular (66 cm)	0.21 ± 0.07	0.34 ± 0.09	<0.001

UDVA = uncorrected distance visual acuity; CDVA = corrected distance visual acuity; UIVA = uncorrected intermediate visual acuity; DCIVA = distance-corrected intermediate visual acuity.

**Table 4:** Binocular distance-corrected defocus results.

Defocus	IsoPure 1.2.3 LogMAR	PCB00 LogMAR	*p* value
+1.00 D	0.23 ± 0.08	0.24 ± 0.09	0.89
Mean ± SD			
+0.50 D	0.10 ± 0.07	0.10 ± 0.08	0.95
Mean ± SD			
0.0 D	0.00 ± 0.01	0.01 ± 0.01	0.91
Mean ± SD			
0.50 D	0.08 ± 0.07	0.10 ± 0.07	0.62
Mean ± SD			
−1.00 D	0.16 ± 0.04	0.20 ± 0.05	<0.05
Mean ± SD			
−1.50 D	0.20 ± 0.07	0.33 ± 0.06	<0.05
Mean ± SD			
−2.00 D	0.38 ± 0.05	0.50 ± 0.07	<0.05
Mean ± SD			

logMAR = logarithm of the minimum angle of resolution.

**Table 5:** Binocular contrast sensitivity.

Spatial frequency	IsoPure 1.2.3 LogCS	PCB00 LogCS	*p* value
1.5	2.00 ± 0.15	1.92 ± 0.11	0.89
3	1.78 ± 0.12	1.80 ± 0.12	0.94
6	1.85 ± 0.09	1.86 ± 0.08	0.95
12	1.44 ± 0.07	1.54 ± 0.10	0.49
18	1.22 ± 0.09	1.29 ± 0.11	0.58

Contrast sensitivity measured with the CSO Tester under photopic conditions at different spatial frequencies (cycles per degree) at 12 months postoperatively. LogCS = logarithm of the contrast sensitivity.

**Table 6:** VF-14 score.

Activities	MPS IsoPure 1.2.3 *N* = 42	MPS PCB00 *N* = 42	*p* value
Playing card games	3.20 ± 0.35	1,42 ± 0,41	<0.05
Cooking	3.76 ± 0.55	2,12 ± 0,49	<0.05
Performance of sporting activities	3.80 ± 0.31	3,61 ± 0,42	0.39
Reading small print	2.10 ± 0.64	1,92 ± 0,52	0.22
Reading books and magazines	2.01 ± 0.56	1,99 ± 0,67	0.24
Reading large print	3.31 ± 0.25	2,32 ± 0,43	<0.05
Distinguishing people close up	3.40 ± 0.35	2,32 ± 0,49	<0.05
Distinguishing traffic signs, names of streets, shop signs	3.27 ± 0.35	3,17 ± 0,41	0.53

MPS: mean point score (4-without difficulties, 0-performance impossible due to difficulties).

## Data Availability

The data used to support the findings of this study are available from the corresponding author upon request.
